# Genome-Wide Characterization and Function Analysis of *ZmERD15* Genes’ Response to Saline Stress in *Zea mays* L.

**DOI:** 10.3390/ijms232415721

**Published:** 2022-12-11

**Authors:** Huaming Duan, Qiankun Fu, Hong Lv, Aijun Gao, Xinyu Chen, Qingqing Yang, Yingge Wang, Wanchen Li, Fengling Fu, Haoqiang Yu

**Affiliations:** Key Laboratory of Biology and Genetic Improvement of Maize in Southwest Region, Maize Research Institute, Ministry of Agriculture, Sichuan Agricultural University, Chengdu 611130, China

**Keywords:** ERD, abiotic stress, tolerance, maize

## Abstract

Early responsive dehydration (ERD) genes can be rapidly induced by dehydration. ERD15 genes have been confirmed to regulate various stress responses in plants. However, the maize ERD15 members have not been characterized. In the present study, a total of five *ZmERD15* genes were identified from the maize genome and named *ZmERD15a*, *ZmERD15b*, *ZmERD15c*, *ZmERD15d*, and *ZmERD15e*. Subsequently, their protein properties, gene structure and duplication, chromosomal location, *cis*-acting elements, subcellular localization, expression pattern, and over-expression in yeast were analyzed. The results showed that the ZmERD15 proteins were characterized by a similar size (113–159 aa) and contained a common domain structure, with PAM2 and adjacent PAE1 motifs followed by an acidic region. The ZmERD15 proteins exhibited a close phylogenetic relationship with OsERD15s from rice. Five *ZmERD15* genes were distributed on maize chromosomes 2, 6, 7, and 9 and showed a different exon–intron organization and were expanded by duplication. Besides, the promoter region of the *ZmERD15s* contained abundant *cis*-acting elements that are known to be responsive to stress and hormones. Subcellular localization showed that ZmERD15b and ZmERD15c were localized in the nucleus. ZmERD15a and ZmERD15e were localized in the nucleus and cytoplasm. ZmERD15d was localized in the nucleus and cell membrane. The results of the quantitative real-time PCR (qRT-PCR) showed that the expression of the *ZmERD15* genes was regulated by PEG, salinity, and ABA. The heterologous expression of *ZmERD15a*, *ZmERD15b*, *ZmERD15c*, and *ZmERD15d* significantly enhanced salt tolerance in yeast. In summary, a comprehensive analysis of *ZmERD15s* was conducted in the study. The results will provide insights into further dissecting the biological function and molecular mechanism of *ZmERD15s* regulating of the stress response in maize.

## 1. Introduction

Abiotic stresses, such as drought, high salinity, heat, and freezing, are common environmental factors that affect crop growth and productivity [1,2,3]. However, due to climate change and the increasing population, there is an urgent need to produce high-yield crops [2]. In order to cope with these stimuli, plants have evolved a series of changes at the morphological, physiological, and molecular levels to balance environmental stress and growth [1,3,4].

Early responsive-to-dehydration (ERD) proteins are rapidly induced in response to dehydration stress and play crucial roles in plant growth and stress response. In *Arabidopsis thaliana*, 16 groups of ERD have been isolated from *Arabidopsis* cDNAs induced by 1 h dehydration treatment [5]. ERD can be induced by multiabiotic stresses and acts as a diverse player in plants. For instance, *Arabidopsis* AtERD2 and AtERD8 and maize ZmERD2 are reported as heat shock proteins (HSPs) that are regulated by drought and abscisic acid (ABA) [5,6]. Maize ZmERD3 and *Arabidopsis* AtERD3 both encode methyltransferase [7,8]. The ERD4 of *Salicornia brachiata*, *Brassica juncea*, and *Arabidopsis* are membrane-bound proteins [8,9,10]. AtERD6 encodes a sugar transporter that can be induced by cold and drought stress [11]. AtERD7 acts as a lipid droplet protein and remodels cell membrane lipid composition during cold stress in *Arabidopsis* [12,13]. AtERD9, AtERD11, and AtERD13 encode glutathione-S-transferase [8,14]. AtERD10 and AtERD14 belong to the type II LEA proteins [15]. AtERD5, AtERD12, and AtERD16 encode proline dehydrogenase, allene oxide cyclase, and ubiquitin 60S ribosomal protein, respectively [8].

In plants, the ERD15s have been reported as transcription factors and can be induced by a diversity of stresses. ERD15 proteins are characterized by a PABP interacting Motif 2 (PAM2), the PAM2-Associated Element1 (PAE1) domain, and an IqQPR sequence at the C-terminal end [16]. In *Arabidopsis*, AtERD15 is highly induced by dehydration and ABA and negatively regulates stomatal aperture, drought, and freezing tolerance but positively improves resistance to the bacterial necrotroph *Erwinia carotovora* subsp. *carotovora* [16,17,18]. On the contrary, some ERD15s function as positive regulators in stress response. The *GmERD15B* of *Glycine max* positively enhances salt tolerance in transgenic soybean by increasing the expression levels of those genes related to ABA-signaling, proline content, catalase-peroxidase, the dehydration response, and cation transport [19]. Another *GmERD15* is induced by ER stress and osmotic stress to activate the expression of N-rich protein (NRP) genes and connect ER stress with the osmotic stress-induced cell death signal [20]. The *VaERD15* of *Vitis amurensis* is upregulated by low-temperature treatment and improves the cold tolerance of transgenic *Arabidopsis* [21]. The expression of *ERD15* from *Solanum lycopersicum*, *Solanum pennellii*, and *Ipomoea batatas* is regulated by NaCl, drought, and ABA [22,23,24]. The overexpression of *SpERD15* in *Solanum pennellii* increases drought tolerance in tobacco [24]. However, little is known about ERD15 in crops.

As one of the most important crops, maize acts as a key factor in the development of the economy and in maintaining food security [25]. So far, *ZmERD2*, *ZmERD3*, and *ZmERD4* have been cloned and confirmed to be regulated by multiple stresses, including drought and salinity [6,7,26]. However, the maize *ZmERD15* remains obscure. In this study, the ZmERD15 family members were identified in the maize genome and characterized for their protein characteristics, gene structure and duplication, chromosomal location, *cis*-acting elements, subcellular localization, expression pattern under osmotic, salt, and ABA treatments. Moreover, their potential roles in regulating tolerance were validated in *Saccharomyces cerevisiae* W303a cells. The study provides insights for further underlying the function of *ZmERD15* in stress response.

## 2. Results

### 2.1. The ZmERD15 Members in Maize

Using AtERD15 as a reference, a total of five *ZmERD15* members were identified from the maize genome and named *ZmERD15a*, *ZmERD15b*, *ZmERD15c*, *ZmERD15d*, and *ZmERD15e*. Five *ZmERD15* genes were distributed on maize chromosomes 2, 6, 7, and 9, of which, *ZmERD15a*, *ZmERD15b*, and *ZmERD15c* were located on chromosomes 2, 6, and 7, respectively. *ZmERD15d* and *ZmERD15e* were distributed on chromosome 9. The coding sequence (CDS) of *ZmERD15a*, *ZmERD15b*, *ZmERD15c*, *ZmERD15d*, and *ZmERD15e* were amplified from the cDNA of maize B73 and sequenced with 480, 342, 477, 417, and 423 bp (in length), encoding 159, 113, 158, 138, and 140 amino acids (aa), respectively, with a molecular weight (MW) ranging from 15.28 to 17.74 KDa. The five ZmERD15 were hydrophilic and unstable proteins with grand average hydropathy (GRAVY) < 0 and instability indices (II) > 40. Only ZmERD15b was neutral, with a theoretical isoelectric point (PI) = 7.73, and the other ZmERD15s were acidic proteins (PI < 7.0) (Table 1). The CDSs and amino acid sequences of the ZmERD15s are listed in Table S1.

### 2.2. Multiple Sequence Alignment and Evolutionary Analysis

**Figure 1:**
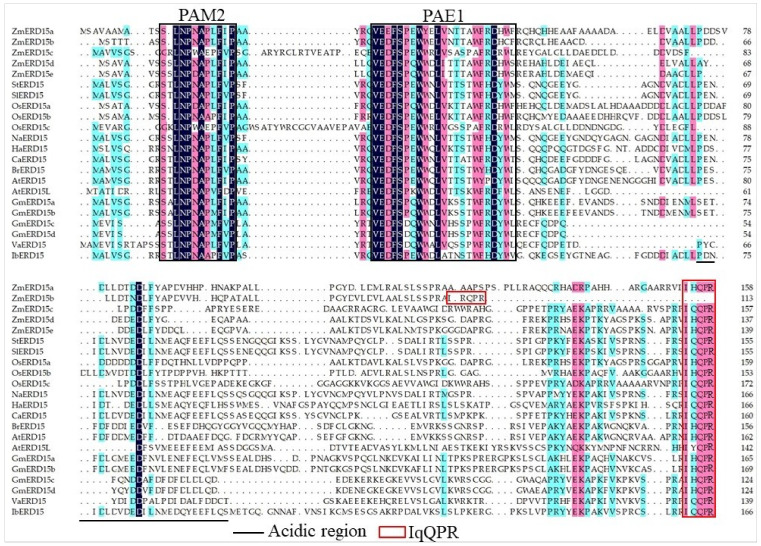
Multiple sequence alignment of ERD15s. The PAM2 and PAE1 are marked by black boxes. The acidic region and IqQPR sequence are marked with black underlining and a red box, respectively. The mazarine, pink, and light-blue backgrounds indicate perfect (100%), high (75%), and low (50%) conservation, respectively.

**Figure 2:**
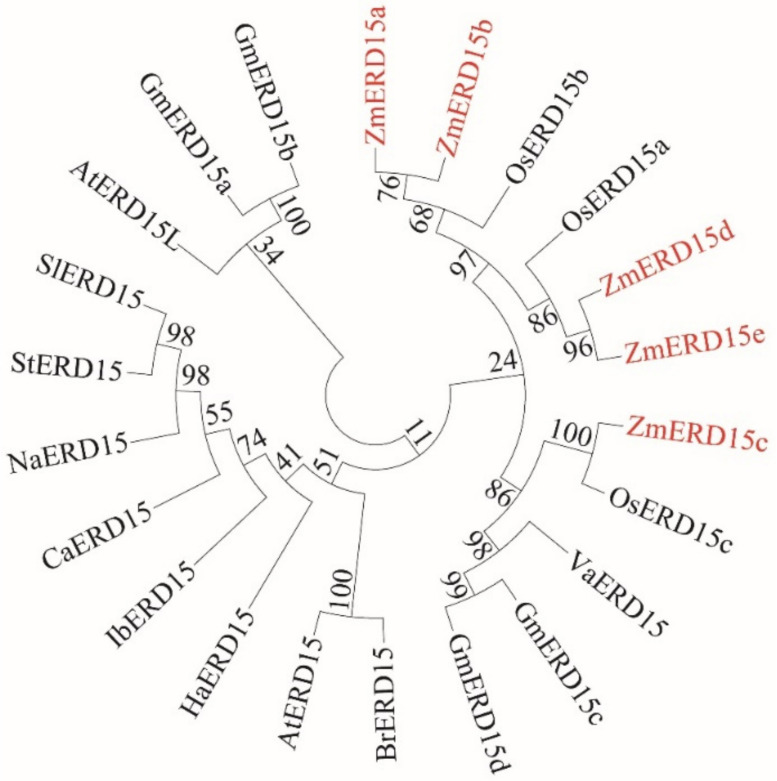
Phylogenetic tree of ERD15s. The ERD15 of *Oryza Sativa* (OsERD15a, Q7XXS2; OsERD15b, Q7EZY8; OsERD15c, Q5W6M4), *Arabidopsis thaliana* (AtERD15, NM_180019; AtERD15L, NM_001340912), *Capsicum annuum* (CaERD15, XP_016571847), *Helianthus annuus* (HaERD15, XP_022036113), *Solanum lycopersicum* (SlERD15, NM_001247532), *Brassica rapa* (BrERD15, NP_001288839), *Nicotiana attenuate* (NaERD15, XP_019228175), *Glycine max* (GmERD15a, XM_006577701; GmERD15b, XM_028335480; GmERD15c, NM_001354922; GmERD15d, XM_028344630), *Vitis amurensis* (VaERD15, JQ687321.1), *Ipomoea batatas* (IbERD15, KF723428.1) and *Solanum tuberosum* (StERD15, XP_006351285) were obtained from NCBI database and used for evolutionary phylogenetic analysis.

In order to explore the sequence features, the protein sequences of the five ZmERD15 were aligned with the ERD15s from other plants. The results showed that all ZmERD15s had a characterized PAM2, PAE1 domain, and an IqQPR sequence at the C-terminal end (Figure 1). The phylogenetic tree exhibited that the ZmERD15s had a higher sequence identity with OsERD15s than those of the dicotyledonous species. The pair of *ZmERD15a* and *ZmERD15b* and the pair of *ZmERD15d* and *ZmERD15e* were genes that were duplicated in maize after the separation of maize and rice. ZmERD15c was most similar to OsERD15c, and the pair ZmERD15a and ERD15b were most similar to OsERD15b, and the pair ZmERD15d and ZmERD15e were most similar to OsERD15a. (Figure 2).

### 2.3. Gene Structure and Motif

**Figure 3:**
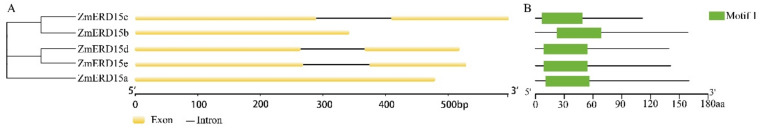
Gene structure (**A**) and motif composition (**B**).

In order to unravel the exon–intron organization of the *ZmERD15* genes, the CDS and genomic DNA (gDNA) of *ZmERD15* were analyzed by the GSDS tool. As shown in Figure 3, the *ZmERD15a* and *ZmERD15b* had one exon but no intron. *ZmERD15c*, *ZmERD15d*, and *ZmERD15e* had one intron and two exons. One conserved motif was predicted in the ZmERD15s and contributed to the composition of the PAM2 and PAE1 domain.

### 2.4. Cis-Acting Elements in ZmERD15s Promoter

In order to better explore the potential function of *ZmERD15s*, the 2000 bp promoter sequence of every *ZmERD15* was analyzed to detect the *cis*-elements by PlantCARE. The results showed that abundant *cis*-elements were predicted in the *ZmERD15s* promoter (Table 2). Among these elements, many TATA-box and CAAT-box elements were found, and they were core elements for the promoter. In addition, many *cis*-elements involved in the stress and hormone response were also revealed. For example, there were 5, 2, 4, 5, and 2 ABRE elements (ABA-responsive elements) in the promoters of *ZmERD15a*, *ZmERD15b*, *ZmERD15c*, *ZmERD15d*, and *ZmERD15e*, respectively, indicating their potential roles in ABA response. Likewise, the MBS element (MYB binding site) involved in the drought response was found in the promoters of *ZmERD15b*, *ZmERD15c*, and *ZmERD15d*, respectively. Therefore, the results suggest that the *ZmERD15* genes possibly participate in regulating plant stress responses.

### 2.5. Subcellular Localization of ZmERD15s

**Figure 4:**
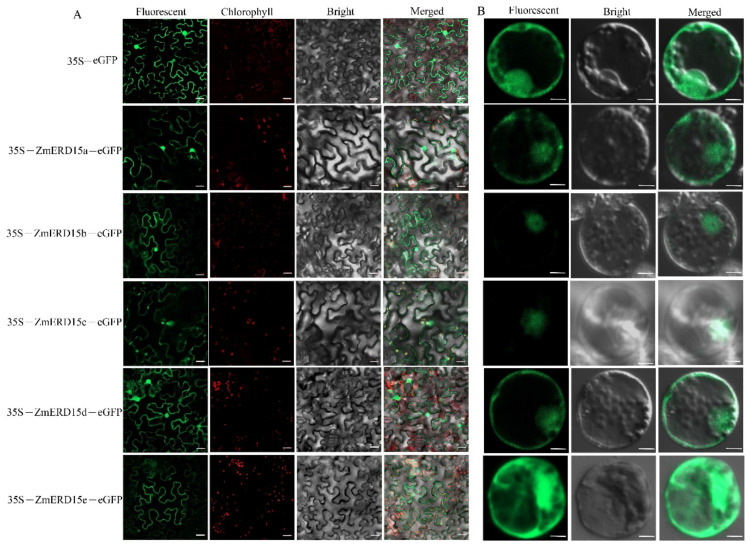
Subcellular localization. (**A**) Localization of ZmERD15s in tobacco leaves. The scale bar is 20 μm. (**B**) Localization of ZmERD15s in maize protoplast. The scale bar is 50 μm. Each ZmERD15 was amplified by PCR without a stop codon and fused to *eGFP* under the control of a 35S promoter. *35S-eGFP* was used as the control.

In order to dissect the subcellular localization of the ZmERD15s, five *ZmERD15* genes were transiently expressed in tobacco leaves. The results showed green fluorescence was observed in the whole cells expressed by the *35S*–*eGFP* plasmid. Similarly, green fluorescence was also observed throughout the cells, including the nucleus, cytoplasm, and cell membrane in the tobacco leaves transformed with each *35S*–*ZmERD15*–*eGFP* (Figure 4A). In order to validate the results, every *35S*–*ZmERD15*–*eGFP* plasmid was transferred into maize protoplasts to express the *ZmERD15*–*eGFP* fusion protein. The results showed that the fluorescence signal of *GFP* was observed in the whole cells, including the nucleus and cytoplasm, expressed by the *35S*–*eGFP*, *35S*–*ZmERD15a*–*eGFP*, and *35S*–*ZmERD15e*–*eGFP* plasmids, respectively. However, the *GFP* signal was only found in the nucleus in the leaves expressed by *35S*–*ZmERD15b*–*eGFP* and *35S*–*ZmERD15c*–*eGFP*, respectively. Moreover, the *ZmERD15d*–*eGFP* protein was localized in the membrane and nucleus (Figure 4B).

### 2.6. Expression Profiles of ZmERD15s under Drought, Salt, and ABA Treatment

**Figure 5:**
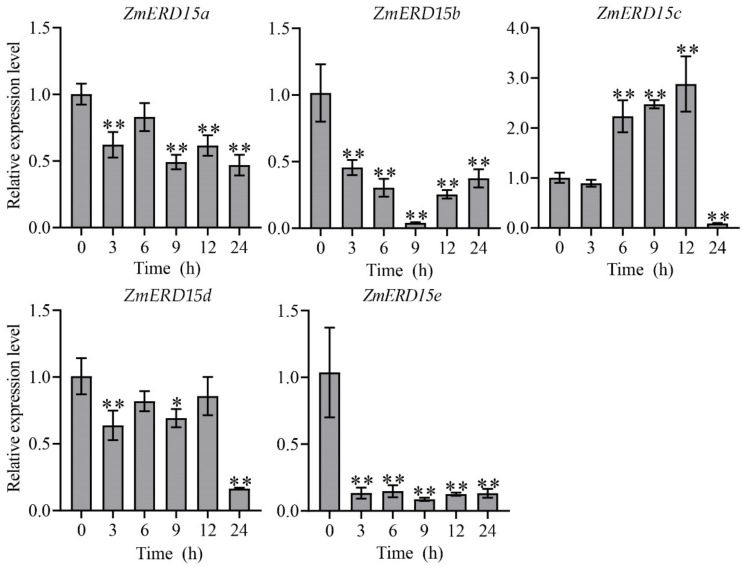
Expression of *ZmERD15s* in response to osmotic stress using 16% PEG-6000. * and ** indicate significant differences from the level of expression at time 0 at *p* < 0.05 and *p* < 0.01, respectively.

**Figure 6:**
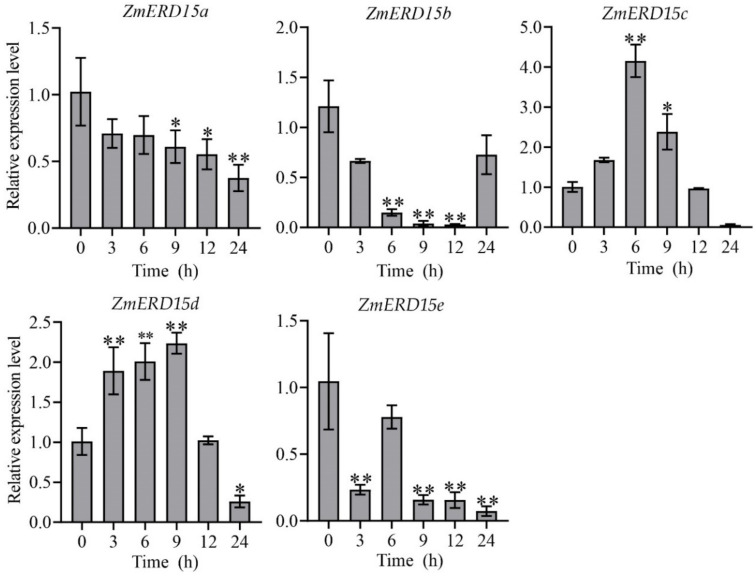
Expression of *ZmERD15s* in response to salinity. The seedlings were subjected to 100 mM NaCl for saline stress. * and ** indicate significant differences from the level of expression at time 0 at *p* < 0.05 and *p* < 0.01, respectively.

**Figure 7:**
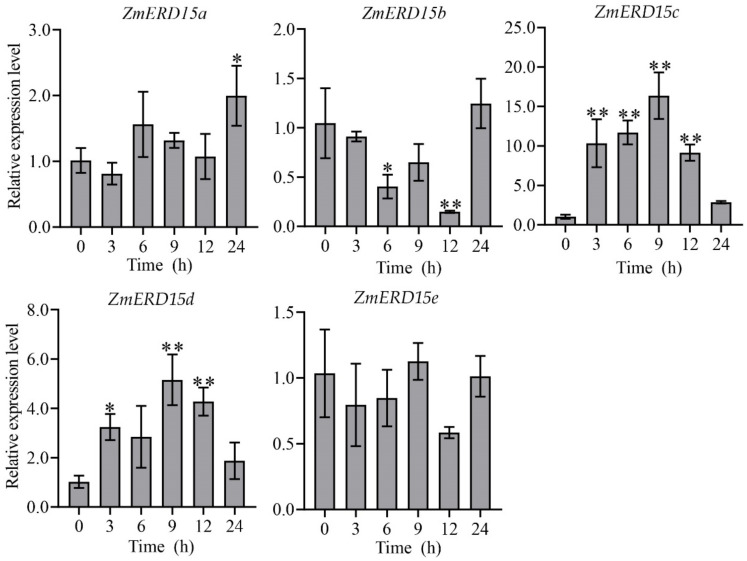
Expression of *ZmERD15s* in response to 100 μM ABA. * and ** indicate significant differences from the level of expression at time 0 at *p* < 0.05 and *p* < 0.01, respectively.

ERD has been proven to be regulated by multiple stresses in previous studies, and gene expression patterns can reveal their potential function. Consequently, the expression of *ZmERD15s* under the osmotic stress of 16% PEG-6000, salinity of 100 mM NaCl, and 100 μM ABA treatment at 0, 3, 6, 9, 12, and 24 h were analyzed by qRT-PCR, respectively. As shown in Figure 5, under drought stress, the expression level of *ZmERD15c* was significantly up-regulated by 16% PEG-6000 by about three-fold compared to the control at 12 h of treatment, although it was inhibited after 24 h of treatment. However, the expression of *ZmERD15a*, *ZmERD15b*, *ZmERD15d*, and *ZmERD15e* was significantly down-regulated by 16% PEG-6000 and reached the lowest level at 24, 9, 24, and 9 h of treatment, respectively. In response to salinity, *ZmERD15c* and *ZmERD15d* showed significantly increased expression under 100 mM NaCl stress, and their expression levels reached a peak at 6 and 9 h of treatment, respectively. The expression of *ZmERD15a*, *ZmERD15b*, and *ZmERD15e* was significantly down-regulated and reached the lowest valley at 24, 12, and 24 h of treatment (Figure 6), respectively. Under the 100 μM ABA treatment, the expression of *ZmERD15a*, *ZmERD15c*, and *ZmERD15d* was significantly up-regulated and reached a peak at 24, 9, and 9 h of treatment, respectively. Whereas the expression of *ZmERD15b* was significantly down-regulated and reached the lowest level at 12 h. Only *ZmERD15e* showed no significant difference compared to the control (Figure 7). These results indicate that *ZmERD15* genes may be involved in stress response via different modes due to their expression diversity.

### 2.7. Heterologous Expression of ZmERD15s Enhances Salt Tolerance in Yeast

**Figure 8:**
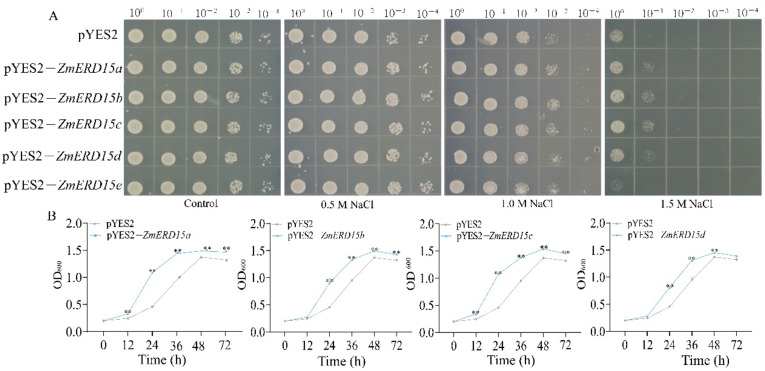
Phenotype of yeast strain expressing *ZmERD15* under salt stress. (**A**) Phenotype of yeast strains on YNBUra− with 2% galactose plates supplemented with 0.5, 1.0, and 1.5 M NaCl, respectively. Photographs were taken after 3 days of incubation at 28 °C in an incubator. (**B**) The growth curve of yeast cells in YNBUra− with 2% galactose liquid medium supplemented with 1.5 M NaCl for 3 days at 28 °C. ** indicates a significant difference at *p* < 0.01.

In order to explore the function of *ZmERD15s* in stress responses, they were over-expressed in the *Saccharomyces cerevisiae* W303a strain for phenotyping under osmotic and salt stresses. The results exhibited that there were no significant differences between the yeast cells expressing pYES2–*ZmERD15* and the empty pYES2 vector under the osmotic stress of 2.0, 2.5, and 3.0 M mannitol (Figure S1). Under salinity stress, the growth of yeast cells was significantly inhibited by 1.0 and 1.5 M NaCl, although there was no difference between the yeast stain carrying pYES2-*ZmERD15* and the empty pYES2 vector on the plates with 0.5 M NaCl. However, the yeast strain transformed by pYES2–*ZmERD15a*, pYES2–*ZmERD15b*, pYES2–*ZmERD15c*, and pYES2–*ZmERD15d* showed higher growth vigor and more colonies than that of the yeast expressing the empty vectors pYES2 and pYES2–Z*mERD15e*, respectively. The yeast carrying pYES2–Z*mERD15e* showed no significant difference compared to the pYES2 control (Figure 8A). Hereafter, the growth curve of the yeast cells harboring pYES2–*ZmERD15a*, pYES2–*ZmERD15b*, pYES2–*ZmERD15c*, and pYES2–*ZmERD15d* during salinity stress using 1.5 M NaCl were monitored. The results showed that the yeast strains with pYES2–*ZmERD15a*, pYES2–*ZmERD15b* pYES2–*ZmERD15c*, and pYES2–*ZmERD15d* exhibited higher growth rates than that of pYES2 between 12 and 24 h of treatment. The OD_600_ of the yeast strains with the *ZmERD15s* was significantly higher than the control after 1.5 M NaCl treatment (Figure 8B). These results confirm that the expression of the *ZmERD15a*, *ZmERD15b*, *ZmERD15c*, and *ZmERD15d* genes enhance the tolerance of yeast to saline stress, respectively.

## 3. Discussion

Environmental stimuli frequently restrict plant growth, development, and productivity [1,2,3]. In order to overcome these stresses, plants have evolved various strategies at the morphological, physiological, and molecular levels, including regulating the expression of the associated stress-responsive genes [1]. In *Arabidopsis*, a certain kind of protein containing 16 groups can be rapidly induced by 1 h dehydration treatment, and they are named early responsive dehydration genes (ERD1 to 16) [5]. Subsequently, ERD families have been confirmed to play crucial roles in stress response, such as drought, cold, and salinity [9,12,18,19]. To date, however, ERD15s have been cloned from a few species, including *Arabidopsis*, *Glycine max*, *Vitis amurensis*, *Solanum lycopersicum*, *Solanum pennellii*, and *Ipomoea batatas* [17,19,20,21,22,23,24]. In addition, other ERD15s in other crop species have not been characterized.

Herein, in the present study, five *ZmERD15* genes were identified from the maize genome and defined as *ZmERD15a*, *ZmERD15b*, *ZmERD15c*, *ZmERD15d*, and *ZmERD15e* (Table 1). The CDSs of the *ZmERD15s* were successfully cloned from the cDNA of maize B73, with lengths of 480, 477, 342, 417, and 423 bp, respectively (Figure S2). They were predicted to be acidic, hydrophilic, and unstable proteins, which was consistent with previous studies [16]. Usually, the ERD15 family proteins are characterized by a similar size (120–170 aa) and common domain structures, with PAM2 and adjacent PAE1 motifs [16]. Here, the ZmERD15s possessed 113–159 aa and shared a similar motif composition with PAM2 and the adjacent PAE1 motifs (Table 1 and Figure 1). The PAM2 motif is a highly conserved amino acid domain that interacts with Poly(A)–binding proteins (PABP), controlling mRNA stability and protein translation [27]. The PAE1 motif is adjacent to PAM2, which is an evolutionarily conserved motif, the function of which is not clear. The acidic region is also found in ZmERD15s and contributes to forming interaction surfaces (Figure 1) [16]. These findings suggest that the five ZmERD15s are typical ERD15s.

Indeed, the ERD15 members can be found in photosynthetic organisms and appear ubiquitous in the plant kingdom, with typically two to three members present in each species [16]. However, we identified five *ZmERD15s* in maize. Gene families generally originate from the same ancestor and produce more copies through duplication [28]. Meanwhile, the *ZmERD15s* showed diversity in their gene structure and were divided into three subgroups (Figure 2 and Figure 3), which was a similar phenomenon found in the previous study and suggested some diversification in the function among the proteins of the ERD15 family, although they were evolutionarily highly conserved [16]. In previous studies, ERD15s were identified as transcription factors to regulate other genes’ expression [8,18,19,21]. In the study, *ZmERD15b* and *ZmERD15c* were only localized in the nucleus in the maize protoplast. In addition to nucleus localization, they were also localized in the cytoplasm and cell membrane in tobacco leaves (Figure 4). It was previously found that soybean *GmERD15* was located in both the cytoplasm and the nucleus and that *AtERD15* did not exhibit transactivation activity or DNA binding activity in yeast [19,29], which does not support its designation as a transcription factor. This is perhaps due to the post-translational modification of ERD15 proteins or the recruiting of interacting factors. The phenomenon is also found in the ZmBES1/BZR1 transcription factor family [30,31].

The stress-associated transcription factors can be activated through signaling transduction and binding to the *cis*-acting elements of downstream gene promoters to regulate their expression. The presence and type of the *cis*-acting elements in the gene promoter region can be used to predict gene expression, which provides information about gene function [32]. In the promoter region of the *ZmERD15* genes, abundant hormone- and stress-responsive *cis*-elements, including MBS and ABRE elements, were predicted (Table 2), indicating that the *ZmERD15* gene might be involved in stress response. The results of the qRT-PCR confirmed that the *ZmERD15* members were regulated by osmotic stress, salinity, and ABA. However, the *ZmERD15s* showed diversity in their response to stress. For instance, the expression of *ZmERD15b* and *ZmERD15c* was significantly up-regulated and down-regulated by osmotic stress and salinity, as well as ABA, respectively (Figure 5, Figure 6 and Figure 7). Furthermore, the over-expression of *ZmERD15a*, *ZmERD15b*, *ZmERD15c*, and *ZmERD15d* significantly improved saline tolerance in yeast, but there was no significant difference under osmotic stress (Figure 8 and Figure S1). It is proposed that ZmERD15s regulate different genes in yeast. Previous studies show that plant transcription factors can enhance stress tolerance in yeast, although there are no homologs in yeast. For instance, the exotic expression of *JrWRKY6* and *JrWRKY53* from *Juglans regia*, *EsDREB2B* from *Eremosparton songoricum*, and *CaCAP2* from *Cicer arietinum* improves multiple abiotic stress tolerances in yeast cells, including saline, osmotic, heat, and cold stresses [33,34,35,36]. In *Arabidopsis*, *AtERD15* is induced by biotic and abiotic factors but acts as a negative regulator in ABA signaling to negatively regulate drought and freezing tolerance [18]. However, *GmERD15*, *VaERD15*, and *SpERD15* from *Glycine max*, *Vitis amurensis*, and *Solanum pennellii* positively regulate salt, cold, and drought tolerance, respectively [19,21,24]. When taken together, the existing data implicate that *ZmERD15s* play crucial roles in stress response via different pathways.

In summary, five *ZmERD15* genes were identified from the maize genome and defined as *ZmERD15a*, *ZmERD15b*, *ZmERD15c*, *ZmERD15d*, and *ZmERD15e*. The ZmERD15 proteins were characterized by a similar size (113–159 aa) and contained a common domain structure with PAM2 and adjacent PAE1 motifs followed by an acidic region. The ZmERD15 proteins exhibited nuclear, as well as other cellular localizations. The five *ZmERD15* genes were distributed on maize chromosomes 2, 6, 7, and 9 and showed different exon–intron organization and were expanded by duplication. Besides, the promoter region of the ZmERD15s contained abundant stress- and hormone-response *cis*-acting elements. The expression of the *ZmERD15s* was regulated by drought, salinity, and ABA. The heterologous expression of *ZmERD15a*, *ZmERD15b*, *ZmERD15c*, and *ZmERD15d* significantly enhanced salt tolerance in yeast, respectively. The study provides insights into revealing the function and molecular mechanism of *ZmERD15s* in stress response in plants.

## 4. Materials and Methods

### 4.1. Plant Materials and Growth Conditions

The seeds of the maize-inbred line B73 were germinated in filter paper and then transplanted into Hogeland nutrient solution for hydroponic culture under 16 h of light at 28 °C and 8 h of dark at 25 °C, as described by Sun et al. [37]. At the three-leaf stage, seedlings of the same size were divided into four groups. The first group of seedlings were used as a control without treatment. The other three groups of seedlings were subjected to osmotic stress, including 16% PEG–6000 and 100 mM NaCl, as well as 100 μ mol/L ABA induction, respectively. At 0, 3, 6, 9, 12, and 24 h of treatment, the leaves were collected, ground in liquid nitrogen, and stored at −80 °C for RNA extraction.

### 4.2. Identification of ZmERD15 Genes in Maize

To identify the *ZmERD15* genes, the maize cDNA and protein database of B73 (Zm-B73-REFERENCE-GRAMENE-4.0) were downloaded from the MaizeGDB database (https://download.maizegdb.org/Zm-B73-REFERENCE-GRAMENE-4.0/, accessed on 5 January 2022). Subsequently, the CDSs and amino acid sequences of the two *Arabidopsis* AtERD15s (AtERD15, AT2G41430; AtERD15L, AT4G14270) and the three rice OsERD15s (OsERD15a, Q7XXS2; OsERD15b, Q7EZY8; OsERD15c, Q5W6M4) (Table S1) were retrieved from the *Arabidopsis* information resource (TAIR) (https://www.arabidopsis.org/, accessed on 5 January 2022) and the NCBI database (https://www.ncbi.nlm.nih.gov/genbank/, accessed on 5 January 2022) [16] and were used as queries to perform local BLASTn and BLASTp (E-value < 1 × 10^–6^) to obtain ZmERD15. The amino acid sequences of the candidates were analyzed by using the hidden Markov model (HMM) profiles of the PAM2 motif (PF07145) from the Pfam database (https://pfam.xfam.org/, accessed on 7 January 2022). After removing the redundant sequences, the candidates were identified as the ZmERD15s and used in subsequent analysis.

### 4.3. Analysis of ZmERD15 Protein Properties

The protein properties of the ZmERD15s, including their molecular weight, instability coefficient, grand average of hydropathicity (GRAVY), and theoretical isoelectric point, were analyzed using ProtParam tool from the Expasy database (https://web.expasy.org/protparam/, accessed on 15 January 2022). The secondary structures of the ZmERD15s were analyzed by using a secondary structure prediction tool provided by PRABI-Lyon-Gerland (https://npsa-prabi.ibcp.fr/cgi-bin/npsa_automat.pl?page=/NPSA/npsa_sopma.html, accessed on 15 January 2022). The conserved domains and motifs were analyzed using the Conserved Domain Database (CDD, https://www.ncbi.nlm.nih.gov/Structure/cdd/wrpsb.cgi, accessed on 15 January 2022) and Multiple EM for motif elicitation (MEME, https://meme-suite.org/meme/doc/meme.html, accessed on 15 January 2022), respectively [38].

### 4.4. Multialignment and Phylogenetic Analysis of the ZmERD15s

The amino acid sequences of the ERD15 of *Oryza Sativa* (OsERD15a, OsERD15b, and OsERD15c), *Arabidopsis thaliana* (AtERD15 and AtERD15L), *Capsicum annuum* (CaERD15), *Helianthus annuus* (HaERD15), *Solanum lycopersicum* (SlERD15), *Brassica rapa* (BrERD15), *Nicotiana attenuate* (NaERD15), *Glycine max* (GmERD15a, GsERD15b, GmERD15c, and GmERD15d), *Vitis amurensis* (VaERD15), *Ipomoea batatas* (IbERD15) and *Solanum tuberosum* (StERD15) were obtained from the NCBI database and used to multialign with the five ZmERD15s using DNAMAN software. The phylogenetic tree was built in MEGA7.0 software using the Neighbor-Joining method with a step size of 1000. The amino acid sequences of these ERD15s are listed in Table S1.

### 4.5. Analysis of Gene Structure and Cis-Elements

The chromosome location of the *ZmERD15* genes was obtained from the maizeGDB database. The CDS and gDNA sequence of the *ZmERD15s* were obtained from maizeGDB and used for exon–intron analysis using Gene Structure Display Server (GSDS, http://gsds.gao-lab.org/, accessed on 5 February 2022). The 2000 bp upstream sequence of the transcription start site (TSS) of every *ZmERD15* was downloaded from maizeGDB and used for *cis*-acting-element analysis using plantCARE (http://bioinformatics.psb.ugent.be/webtools/plantcare/html/, accessed on 10 February 2022) [39].

### 4.6. Subcellular Localization of ZmERD15s

The specific primers (Table S2) were designed using Primer 5, synthesized at TSINGKE (Chengdu, China), and used to amplify the CDS of each *ZmERD15* from B73 cDNA by PCR amplification. The stop codon in each reverse primer was removed. The *pCAMBIA2300*-*35S*-*eGFP* plasmid was linearized using *BamHI* and *XbaI* (TaKaRa, Dalian, China). The PCR product of each full length of the CDS of *ZmERD15* was cloned into linearized *pCAMBIA2300*-*35S*-*eGFP* using the ClonExpress^®^ II One Step Cloning Kit (Vazyme, Nanjing, China) to generate the *35S*-*ZmERD15*-*eGFP* plasmid. Hereafter, every *35S*-*ZmERD15*-*eGFP* construct was transferred into *Agrobacterium* GV3101 and used for transforming the tobacco (*Nicotiana benthamiana*) leaves by infiltration, according to the method described by Sun et al. [37]. After injection, the tobacco seedlings were cultured for 36–48 h at 28 °C in the dark; then, the leaves were collected and used for imaging fluorescence by a confocal microscope LSM800 (Carl Zeiss, Oberkochen, Germany).

Meanwhile, the maize protoplasts were isolated from the etiolated maize seedlings, as described by Fu et al. [40], with minor modification. The leaves were cut into thin strips of about 0.5 mm and enzymatically hydrolyzed for 6 h at 28 °C. The protoplasts were collected by centrifuging at 150× *g*, resuspended using a W5 solution (154 mM NaCl, 125 mM CaCl_2_, 5 mM KCl and 2 mM MES, pH 5.7), diluted to 2 × 10^5^ cells/mL using MMG solution (15 mM MgCl_2_, 0.4 M mannitol and 4 mM MES, pH 5.7), and used for transiently expressing the *35S*-*ZmERD15*-*eGFP* and *35S*-*eGFP* plasmids (control), respectively. Subsequently, the protoplasts were incubated at 28 °C in the dark for 12–16 h and used for GFP fluorescence imaging using a confocal microscope LSM800 (Carl Zeiss, Oberkochen, Germany).

### 4.7. RNA Extraction and qRT-PCR

Total RNA was extracted and reversely transcribed into cDNA by using RNAiso Plus kit (TaKaRa, Dalian, China) and PrimeScript RT reagent Kit with gDNA Eraser (TaKaRa, Dalian, China), respectively. The specific primers were designed using the Primerblast (https://www.ncbi.nlm.nih.gov/tools/primer-blast/, accessed on 5 April 2022), synthesized at TSINGKE (Chengdu, China), and used to perform qRT-PCR (Table S2). The *ZmEFLA* was also amplified by its specific primers (Table S2) and used as a reference gene. The qRT-PCR was performed using the SYBR Green I kit (TaKaRa, Dalian, China) in the CFX-96 system (Bio-Rad, Hercules, CA, USA) as described by Yu et al. [30]. The relative quantification of each *ZmERD15* was calculated by the 2^−ΔΔCT^ method [41]. The data are presented as the mean values ± standard deviation (SD). The statistical significance among three biological replicates was tested by Student’s *t*-tests.

### 4.8. Yeast Expressing Vector Construction and Transformation

The specific primers were designed (using primer 5), synthesized, and used to amplify the CDS of each *ZmERD15* (Table S2). The pYES2 plasmid was linearized using *Bam*HI and *Xho*I (TaKaRa, Dalian, China). The B73 cDNA was used as the template for PCR amplification. The PCR products were subcloned into pYES2 to create the pYES2-*ZmERD15* plasmid. Each pYES2-*ZmERD15* and pYES2 empty vector was transferred into *Saccharomyces cerevisiae* W303a cells via the lithium acetate method [42]. After transformation, the yeast cells were spread on yeast nitrogen plates lacking uracil (YNBUra−) and were cultured for 2–3 days at 28 °C for screening the positive transformants. Subsequently, the positive transformants of each *ZmERD15* were transferred into YNBUra− with 2% galactose liquid medium and incubated overnight to an OD_600_ ≈ 1.0. The cultures were diluted to 10^0^, 10^−1^,10^−2^,10^−3^, and 10^−4^ using sterilized water. Thereafter, 8 μL of each diluted solution was spotted onto YNBUra− with 2% galactose solid medium containing 0.5, 1.0, 1.5 M NaCl and 2.0, 2.5, 3.0 M mannitol, respectively, and incubated for 2–3 days at 28 °C and used for phenotyping. Moreover, the 1 mL yeast cells with an OD_600_ ≈ 0.2 were added into 30 mL liquid YNBUra− with 2% galactose, with 1.5 M NaCl, and incubated at 28 °C. At 0, 12, 24, 36, 48, and 72 h, the OD_600_ was measured and used for monitoring the growth curve of the three replicates. The pYES2 vector was used as a control. The 2% galactose was used to induce the expression of the *ZmERD15s* under the control of the galactose gene promoter.

## Figures and Tables

**Table 1:** The ZmERD15 members in maize.

Gene ID	Gene Name	Chromosome Distribution	CDS (bp)	Protein Properties					Second Structure (%)		
				Number of aa	MW (KDa)	PI	GRAVY	II	α-Helix	β-Turn	Random Coil
Zm00001d007097	*ZmERD15a*	2	480	159	17.64	5.94	−0.351	53.06	45.91	6.29	37.11
Zm00001d022416	*ZmERD15b*	7	342	113	16.25	7.73	−0. 635	65.44	47.18	7.75	37.32
Zm00001d038003	*ZmERD15c*	6	477	158	17.74	5.04	−0. 751	56.70	35.44	5.70	50.00
Zm00001d047470	*ZmERD15d*	9	417	138	15.28	5.47	−0.446	51.86	45.65	2.90	49.28
Zm00001d048165	*ZmERD15e*	9	423	140	15.46	4.97	−0.562	47.70	61.64	0	38.36

**Table 2:** The *cis*-acting elements of *ZmERD15s* promoter region.

*Cis*-Acting Elements	Function	The Number of *cis*-Elements				
		*ZmERD15a*	*ZmERD15b*	*ZmERD15c*	*ZmERD15d*	*ZmERD15e*
TATA-box	core promoter element around −30 of transcription start	54	53	22	29	16
CAAT-box	common cis-acting element in promoter and enhancer regions	14	7	12	15	7
TC-rich repeats	defense and stress responsiveness	1	1	0	0	0
MBS	MYB binding site involved in drought-inducibility	0	3	1	1	0
LTR	low-temperature responsiveness	0	0	2	0	0
GC-motif	anoxic specific inducibility	2	0	0	0	1
ARE	anaerobic induction	2	3	0	0	5
ABRE	ABA-responsive	5	2	4	5	2
CGTCA-motif	MeJA-responsiveness	3	1	6	1	2
TGACG-motif		3	1	6	1	2
TCA-motif	salicylic acid responsive	0	1	0	0	0
TGA-element	Auxin-responsive element	2	2	1	1	0

## Data Availability

Not applicable.

## Supplementary Materials

The following supporting information can be downloaded at: https://www.mdpi.com/article/10.3390/ijms232415721/s1.
