# Supplementary material for: Genome-Wide Characterization and Function Analysis of ZmERD15 Genes’ Response to Saline Stress in Zea mays L
Source: Int J Mol Sci. 2022 Dec 11;23(24):15721. doi: 10.3390/ijms232415721 (PMC9779859; doi:10.3390/ijms232415721)

Figure S1. Phenotype of yeast under drought stress mimicking by mannitol.

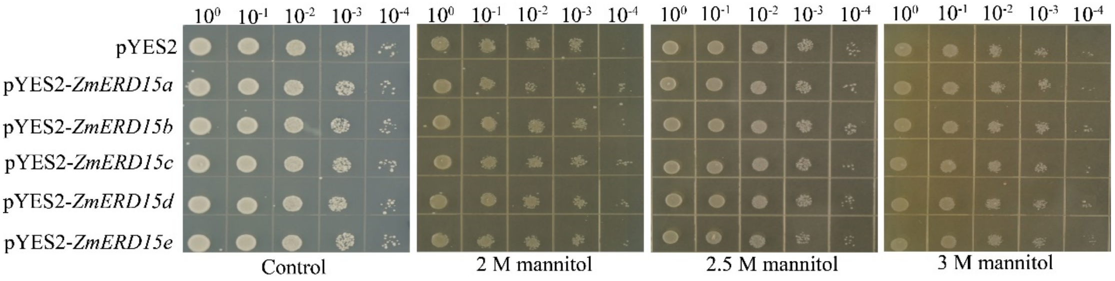

Figure S2. The amplified fragments of ZmERD15s by PCR.

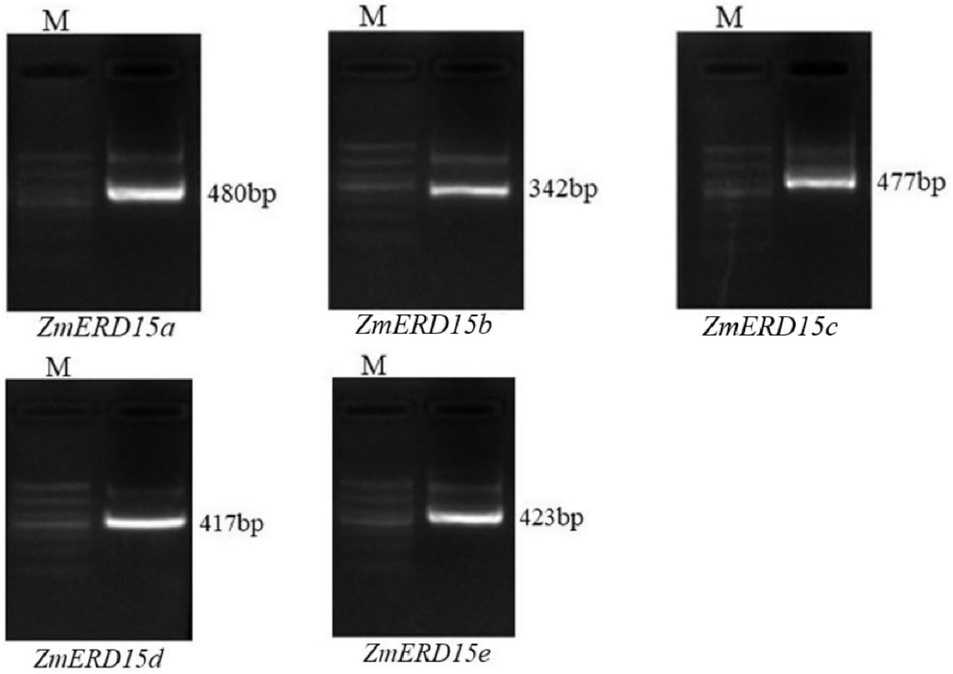

Supplement: Supplementary file 1 [file ijms-23-15721-s001.zip › Figure S1- S2.pdf]
