# Supplementary material for: Genome-Wide Characterization and Function Analysis of ZmERD15 Genes’ Response to Saline Stress in Zea mays L
Source: Int J Mol Sci. 2022 Dec 11;23(24):15721. doi: 10.3390/ijms232415721 (PMC9779859; doi:10.3390/ijms232415721)
Supplement: Supplementary file 1 [file ijms-23-15721-s001.zip › Table S2.pdf]

Table S1. Primers used in this study

Gene	Primer sequence (5′-3′)	Primers used
ZmERD15a	ggacagggtacccggggatccATGAGCGCCGTGGCCGCC<br>ggtactagtgtcgactctagaGCGAGGCTGGTGGATGATG	Primers for subcellular location
	ATGGCAAGCAGCCTGATCTT<br>GCATCCTGGAGTCGTTGACA	Primers for qRT-PCR
	cttggtaccgagctcggatccATGAGCGCCGTGGCCGCC<br>ccctctagatgcatgctcgagGCGAGGCTGGTGGATGATG	Primers for pYES2-*ZmERD15a* construction
ZmERD15b	ggacagggtacccggggatccATGGCGGTTGTGAGCGGC<br>ggtactagtgtcgactctagaGCGCGGCTGCTGGATGGG	Primers for subcellular location
	CAGTGTCACATCTTGGCACG<br>AGCCCAGATCCAGAAACTGC	Primers for qRT-PCR
	cttggtaccgagctcggatccATGGCGGTTGTGAGCGGC<br>ccctctagatgcatgctcgagGCGCGGCTGCTGGATGGG	Primers for pYES2-*ZmERD15b* construction
ZmERD15c	ggacagggtacccggggatccATGAGCACCACAACGGCG<br>ggtactagtgtcgactctagaGCGAGGCTGGCGGATGGC	Primers for subcellular location
	TCTCGGCAGCCACAAGAAAC<br>TAGAACACAGACAGGCACGG	Primers for qRT-PCR
	cttggtaccgagctcggatccATGAGCACCACAACGGCG<br>ccctctagatgcatgctcgagGCGAGGCTGGCGGATGGC	Primers for pYES2-*ZmERD15c* construction
ZmERD15d	ggacagggtacccggggatccATGAGTGCCGTCGCGGTT<br>ggtactagtgtcgactctagaGCGAGGCTGGTGGATCACG	Primers for subcellular location
	CGTCGTGGTCTGTCTGTCAA<br>CACGAGGAGAGCAGCAGTAA	Primers for qRT-PCR
	cttggtaccgagctcggatccATGAGTGCCGTCGCGGTT<br>ccctctagatgcatgctcgagGCGAGGCTGGTGGATCACG	Primers for pYES2-*ZmERD15d* construction
ZmERD15e	ggacagggtacccggggatccATGAGCGCCGTGGCGGTT<br>ggtactagtgtcgactctagaGCGAGGCTGGTGGATCACG	Primers for subcellular location
	CCTACGCCGACACGTTTTCC<br>AGTGAGTGAGCTTCTGGTGC	Primers for qRT-PCR
	cttggtaccgagctcggatccATGAGCGCCGTGGCGGTT<br>ccctctagatgcatgctcgagGCGAGGCTGGTGGATCACG	Primers for pYES2-*ZmERD15e* construction
Zm-EFLA	TGGGCCTACTGGTCTTACTACTGA<br>ACATACCCACGCTTCAGATCCT	Reference gene for qRT-PCR
